# The Effect of Chronic Candesartan Therapy on the Metabolic Profile and Renal Tissue Cytokine Levels in the Obese Zucker Rat

**DOI:** 10.1155/2010/841343

**Published:** 2010-05-17

**Authors:** Carolyn M. Ecelbarger, Arjun Rash, Rajesh K. Sinha, Swasti Tiwari

**Affiliations:** ^1^Division of Endocrinology and Metabolism, Department of Medicine, Georgetown University, Washington, DC 20057, USA; ^2^Laboratory of Immunoregulation, National Institute of Allergy and Infectious Disease, National Institutes of Health, Bethesda, MD 20892, USA

## Abstract

The effect of candesartan, an angiotensin-II type-1 receptor antagonist, on the metabolic profile and renal inflammation is unclear. We evaluated this relationship by feeding male lean (LZ) and obese (OZ) Zucker rats chow or chow with candesartan (23.5 mg/kg · diet) for 14 weeks (*n* = 6–8/treatment/body type). Candesartan reduced serum triglycerides, plasma creatinine, urine albumin, and renal cortical collagen and glycogen deposition in the OZ. An ELISA-based cytokine array revealed that candesartan normalized elevated renal interleukin (IL) 1-*β* and monocyte chemoattractant protein-1 (MCP-1) levels in OZ. Nonetheless, candesartan impaired glucose tolerance, and did not lower blood insulin or glucose levels. Moreover, renal IL-1*α*, -2, -4, -6 and -10 tumor necrosis factor-*α*, interferon-*γ*, were significantly reduced in OZ relative to LZ, and increased by candesartan. Furthermore, candesartan increased growth-regulated oncogene, transforming growth factor-*β*1 and IL-18 in OZ kidneys to a level higher than LZ or untreated OZ. Candesartan did not affect renal cytokine levels in LZ. Overall, candesartan attenuated renal disease and improved renal function in OZ, despite mixed effects on metabolic factors and cytokines. Reduced plasma triglycerides and/or renal MCP-1 and IL-1*β* may have had a role in this protection. However, these effects were clearly independent of any improvement in glucose tolerance.

## 1. Introduction

Renal disease or nephropathy is a frequent complication of the metabolic syndrome and a leading cause of end-stage renal failure in type II diabetes [1]. Hypertension, inflammation, insulin resistance, and/or an altered metabolic profile, including poor glycemic control and dyslipidemia are among the several mechanisms or “risk factors” associated with this disorder [2–9]. Elucidation of these mechanism(s) is crucial in guiding the development of more efficacious therapies to combat renal disease, improve the quality of life in this patient population, and assuage the societal burden of the metabolic syndrome. 

Angiotensin II (Ang II), a potent vasoconstrictor, and mediator of oxidative stress and proliferative pathways in tissue, is a strong candidate in modulating many of these risk factors [10–12]. Ang II raises blood pressure [13], induces insulin resistance [14, 15], and increases inflammation by either directly activating immune cells or by producing inflammatory mediators [16, 17]. Furthermore, clinical as well as basic research studies have shown that treatment with any of several available Ang II receptor blocker (ARBs), compounds that bind antagonistically to the Ang II, type I receptor (AT1R), results in renoprotection [18–21]. Candesartan (CAN), one such ARB, commonly prescribed to lower blood pressure (BP), has clearly been shown to improve renal function and attenuate renal disease [20, 21]; however the mechanism(s) underlying this protection is not entirely clear. 

The obese Zucker rat is a model for human metabolic syndrome with associated renal disease [22, 23]. One manner in which CAN would be expected to exert significant reno-protection is by lowering BP. We have already published BP in response to chronic CAN therapy in the same set of rats [24]. CAN treatment for 14 weeks resulted in a marked and sustained fall in BP of approximately 20–30 mm Hg, in both lean and obese rats [24]. ARBs have also been demonstrated to reduce inflammation in tissues, such as the pancreas, heart, brain, vasculature, and adrenal gland [25–27], improve insulin sensitivity [28–30], and activate PPAR-*γ*, an intracellular nuclear hormone receptor involved in the regulation of carbohydrate and lipid metabolism [31]. Therefore, the beneficial actions of ARBs on the kidney may extend well beyond their BP-lowering actions. 

 In this report, we examine the effects of chronic CAN therapy on renal function and disease in obese and lean Zucker rats. We determine whether attenuation of renal inflammation and/or factors associated with the metabolic syndrome may contribute to any observed renoprotective effects of CAN. We utilize a cytokine array to measure 14 cytokines/chemokines in the whole kidney homogenates of treated rats. In addition, we measure certain indices of the metabolic profile including plasma triglycerides and glucose tolerance. We hypothesize that chronic CAN therapy will reduce renal inflammation (perhaps via improvement in the metabolic status), in the obese Zucker rat, thus potentially contributing to attenuation of renal disease in these rats.

## 2. Methods

### 2.1. Animals Study Design

Thirty-two male Zucker rats (16 lean and 16 obese) were obtained from Charles River Laboratories (Wilmington, MA). Rats were singly housed in microfilter top, plastic cages with a normal 12-hour light/dark cycle according to protocols approved by the Georgetown Animal Care and Use Committee, an AAALAC (Association for Assessment and Accreditation of Laboratory Animal Care, International) approved facility. At about 9 weeks of age, 8 rats from each body type were randomly assigned to either ground control diet (Purina 5001 Rodent Chow, Purina Mills, St. Louis, MO) solidified in agar with 70% water, or the same base diet with 23.5 mg candesartan cilexetil (Atacand, AstraZeneca Pharmaceuticals, Wilmington, DE) incorporated per kg diet (wet weight). This resulted in an approximate dose of 3-4 mg/kg · bw/day of CAN in the treated rats. Rats were weighed weekly and fed diets and received water *ad libitum* for 14 weeks. Urine was collected at 12 weeks in metabolic cages.

### 2.2. Glucose-Tolerance Test

 A glucose tolerance test (GTT) was given to all rats at 13 weeks to assess their ability to rapidly regulate blood glucose (a function of insulin sensitivity). The test was performed as described previously [32, 33]. Briefly, rats were given a 50% dextrose solution (3 ml/kg · bw) intraperitoneally. Glucose was measured in tail blood with a glucometer (One-Touch, Lifescan, Johnson & Johnson) after pricking the tail at 15, 30, 60, 90, and 120 minutes postglucose administration. Blood glucose concentration over time was plotted and the areas under the curves were calculated and statistically compared.

### 2.3. Kidneys and Blood Collection

 At the end of the 14 weeks, rats were deeply anesthetized with sodium pentobarbital and the right kidney perfusion fixed, as described previously [34, 35], for histochemical analyses. Prior to perfusion, some blood was collected into both K_3_-EDTA- and Na^+^-heparin-containing vacutainer tubes (Becton-Dickinson, Franklin Lakes, NJ). Immediately after euthanization, the left kidney was removed and processed as a whole kidney homogenate for the analysis of protein levels.

### 2.4. Plasma and Urine Analyses

 Plasma insulin levels were analyzed in blood collected at euthanization by a radioimmunoassay, as previously described [36]. Triglycerides and urine albumin were analyzed by colorimetric assays (Sigma, St. Louis, MO and Exocell, Philadelphia, PA, resp.). Plasma creatinine was determined by the Jaffe rate method (Creatinine Analyzer 2, Beckman Diagnostics Systems Group, Brea, CA).

### 2.5. Histochemical Staining

After fixation with 4% paraformaldehyde, the right kidney was processed to paraffin, sectioned at 4 *μ*m, and stained with periodic acid-Schiff's (PAS; for demonstration of glycogen deposition) to determine glomerulosclerosis, which is defined as thickening of the basement membrane and mesangial expansion) or Masson's trichrome (for demonstration of collagen deposition) to determine tubulointerstitial fibrosis, which is defined as tubular atrophy or dilatation, deposition of extracellular matrix, and interstitial fibroblast proliferation [37].

### 2.6. Cytokine Profile Using an ELISA-Based Cytokine Array

Levels of monocyte chemoattractant protein-1 (MCP-1), interleukins (IL)-1*β*, -1*α*, -2, -6, -5, -4, -10, -18, -12p70, tumor necrosis factor-*α* (TNF-*α*), interferon-*γ* (INF-*γ*), growth-regulated oncogene (GRO-KC), and granulocyte macrophage colony-stimulating-factor (GM-CSF) were determined in the whole kidney homogenates using a rat cytokine/chemokine LINCOplex premixed 96-well plate assay (Millipore, St. Charles, MO, catalog no. RCYTO-80K-PMX).

### 2.7. Western Blotting

Western blotting was performed as previously described [32] on whole kidney homogenates to evaluate the effects of body type and therapy on endothelial nitric oxide synthase (eNOS, NOS3) and TGF-*β*1 (transforming growth factor using commercially available antibodies: polyclonal NB100-91995 (TGF-*β*1, Novus Biologicals, Littleton, CO), and monoclonal 610297 (eNOS, Transduction Laboratories, San Diego, CA).

### 2.8. Statistics

To determine the overall effects of CAN treatment and body type on variables of interest, data were analyzed by two-way (body type × treatment) analysis of variance (ANOVA). The difference between individual pairs of means was analyzed by unpaired *t*-test. If data were nonparametric, not normally distributed, or variances were different, we used the Mann-Whitney rank sum test (Sigma Stat, Chicago, IL). *P* < .05 was considered significant for all analyses. Among the 14 cytokines analyzed in the array, 6, that is, IL-1*α*, IL-4, IL-10, IL-12p70, IFN-*γ* and TNF-*α*, were below detection levels in the untreated obese group, as well as in some treated obese rats (1 or 2 out of 6). Thus the statistical analyses for these 6 cytokines were done in a different way; that is, cytokine levels were categorized (one category was “undetectable”), and a *t*-test (Rank Test) on categorical assignments was done. The other 8 cytokines were analyzed by standard unpaired *t*-test as continuous variables.

## 3. Results

### 3.1. Chronic Candesartan Treatment Improves the Indices of Renal Function and Reduces Pathology

**Figure 1:**
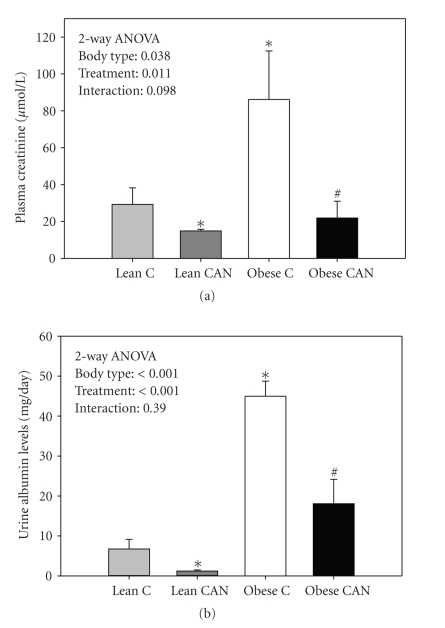
Plasma creatinine (a) and urinary albumin excretion (b) in candesartan-treated (CAN) or -untreated (c) lean and obese rats at the end of 14 weeks of treatment (*n* = 8 per body type/treatment). Obese had significantly increased albumin excretion and higher plasma creatinine relative to lean groups; CANtreated groups were significantly different from control by 2-way ANOVA (*P* < .05). ∗ indicates a significant difference (*P* < .05) from lean control mean and # from obese control mean by unpaired *t*-test.

Obese Zucker rats had significantly higher levels of plasma creatinine (Figure 1(a)) and urine albumin excretion (Figure 1(b)), relative to lean age-mates, indicating impaired renal function and advancing renal disease. Both plasma creatinine and urine albumin were markedly reduced in the obese rats treated with CAN. CAN also resulted in a significant reduction in these two parameters in the lean rats, although the reduction was of considerably lower magnitude. 

**Figure 2:**
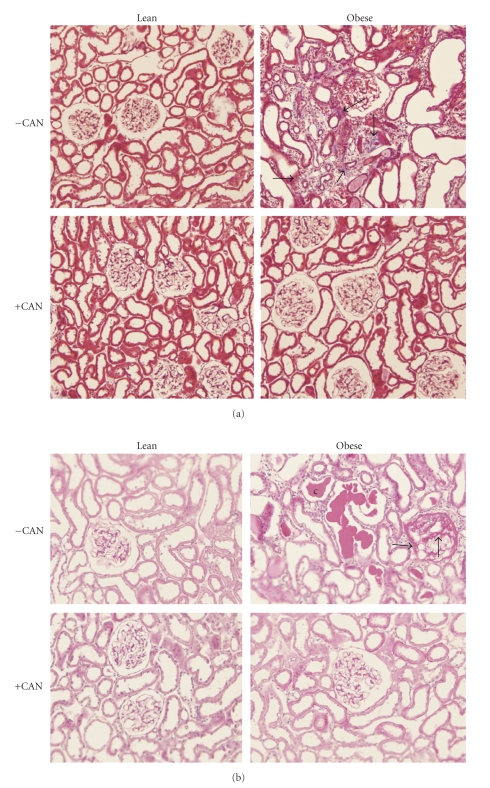
Renal cortical pathology: glomerulosclerosis and tubulointerstitial fibrosis were assessed in periodic acid Schiff and Masson's trichrome-stained paraffin sections (4 *μ*m sections, 6 sections/kidney were analyzed) from Zucker rats (Lean or obese) treated with (+CAN) or without candesartan (−CAN) (*n* = 3/bodytype/treatment). (a) Masson trichrome-stained paraffin sections showing enlarged lumens of the renal tubules and heavy deposition of collagen in the interstitial spaces (light-blue staining, arrows) in untreated rats only. (b) Periodic acid Schiff's-stained paraffin sections. The mesangial expansion is shown by arrows and hyaline casts by (c) in the renal tubules in untreated rats only.

In addition to improving renal function, chronic CAN therapy attenuated renal pathology in obese rats as revealed by histochemical staining. Untreated obese rats demonstrated marked renal pathology as indicated by features of glomerulosclerosis and tubulointerstitial fibrosis in their kidney sections relative to lean rats (Figure 2). Chronic CAN attenuated these pathological features. As shown in Figure 2, Masson trichrome-stained paraffin sections revealed heavy deposition of collagen in the interstitial spaces (light-blue staining, Figure 2(a) and enlarged lumens of the renal tubules in untreated obese rats only. Similarly, mesangial expansion (arrows) and hyaline casts in the renal tubules were found only in untreated obese rats' kidney by periodic Schiff's staining (Figure 2(b)). There was no apparent effect of the chronic CAN therapy on renal histology in the lean rats.

### 3.2. Candesartan Has an Opposite Effect in Lean and Obese Rats with Regard to Water Intake and Urine Volume

 As expected, obese rats gained significantly more weight by the end of the study (Table 1). CAN treatment did not significantly affect weight gain, although there was a trend for weight gain to be less in the obese CAN rats. Absolute water intake and urine volumes were higher in obese rats; however, when normalized by kidney weight, urine volume was lower in obese control rats and corrected (to lean levels) by CAN. In contrast, CAN significantly decreased urine volume in lean rats, so that there was a significant interaction between terms in the 2-way ANOVA. There was also a strong trend for decreased water intake in these treated lean rats (*P* = .064, unpaired *t*-test between Lean Control and Lean CAN).

### 3.3. Candesartan Reduced Serum Triglyceride Levels

Serum triglycerides were significantly increased in obese rats related to lean (Table 1). Candesartan treatment significantly reduced triglyceride levels in obese rats (to a level only 46% of the untreated obese level), and to more modest extent in the lean (to 75% of the untreated lean level). Levels in treated obese rats were still on average 85% higher than lean control.

### 3.4. Candesartan Has an Opposite Effect in Lean and Obese Rats with Regard to Glucose Tolerance

**Figure 3:**
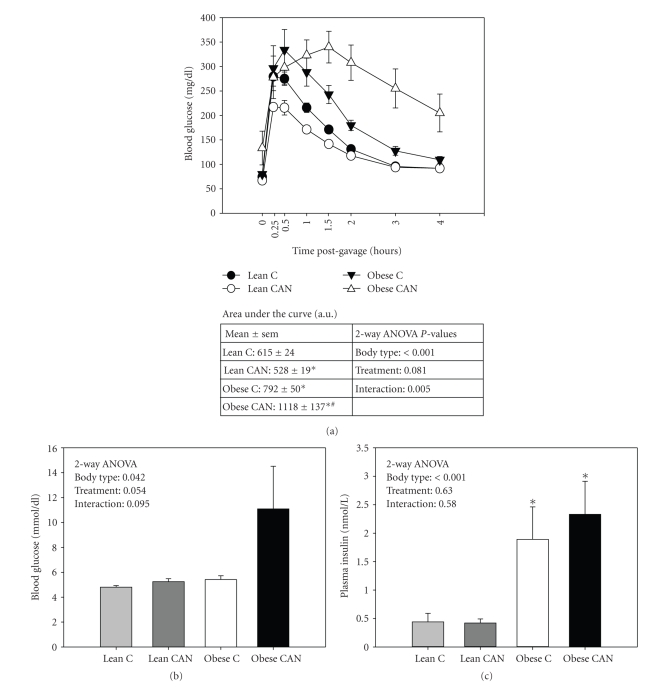
Metabolic function: (a) Glucose-tolerance test (GTT) performed in candesartan-treated (CAN) or untreated (c) lean and obese rats at 13 weeks of treatment (*n* = 8 per body type/treatment). Blood glucose levels measured at different time points in response to 50% dextrose solution (3 ml/kg · bw) given intraperitoneally. Area under the curve was higher for the obese rats compared to the lean, and a significant interaction was found between body type and treatment by 2-way ANOVA (*P* < .05). (b) Blood glucose and (c) plasma insulin levels at the end of 14 weeks of treatment (*n* = 8 per body type/treatment) were different between the body types by 2-way ANOVA (*P* < .05); 14 weeks of CAN treatment did not affect these levels. ∗ indicates a significant difference (*P* < .05) from lean control mean and # from obese control mean, by unpaired *t*-test.

 Obese rats demonstrated significantly slower plasma glucose clearance (glucose tolerance, measured at 13 weeks), in response to intraperitoneally administered glucose (3 ml/kg*·*bw), relative to lean rats (Figure 3(a)). Unexpectedly, CAN treatment further worsened this impairment, as demonstrated by significantly higher area under the curve (AUC) for plasma glucose levels over time in CAN-treated obese rats relative to untreated obese. In lean rats, however, CAN significantly improved glucose tolerance. This led to a significant interaction between body type and treatment by 2-way ANOVA.

In addition, final blood glucose levels (measured just prior to euthanization at 14 weeks) trended toward being higher in obese rats treated with CAN relative to all other groups (Figure 3(b)), *P*-value = .07, as compared to lean control). Plasma insulin levels (Figure 3(c)) were significantly higher in the obese rats, and not altered by CAN in either lean or obese.

### 3.5. Effects of Long-Term Candesartan Treatment on the Cytokine Profile in Kidney Tissue

**Figure 4:**
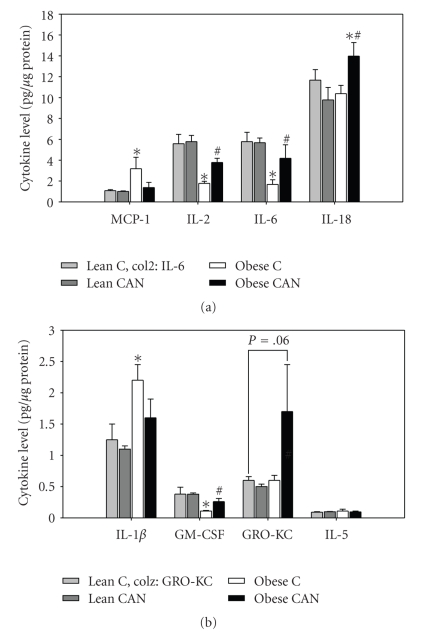
Mean kidney cytokine levels (all rats detectable) (a) higher level cytokines including: monocyte chemotactic protein-1 (MCP1), interleukin-2 (IL-2), interleukin-6 (IL-6), and interleukin-18 (IL-18); (b) lower level cytokines including: interleukin 1*β*(IL-1*β*),granulocyte macrophage colony-stimulating-factor (GM-CSF), growth regulated oncogene (GRO-KC), and interleukin-5 (IL-5) in whole kidney tissue homogenate from candesartan treated (CAN) or untreated (C) lean and obese rats at the end of 14 weeks of treatment (*n* = 8 per body type/treatment). ∗ indicates a significant difference (*P* < .05) from lean control mean and # from obese control mean, by unpaired *t*-test.

 Renal levels of only two cytokines (out of 14) were elevated in obese versus lean rats in the control state, that is, MCP-1 and IL-1*β* (Figures 4(a) and 4(b))*.* Their levels were, respectively.  200% and 70% higher than untreated lean controls. Long-term CAN treatment reduced these levels in obese rats such that they were no longer significantly different than lean. There was no significant effect of CAN on the level of these two cytokines in the lean rats. 

**Figure 5:**
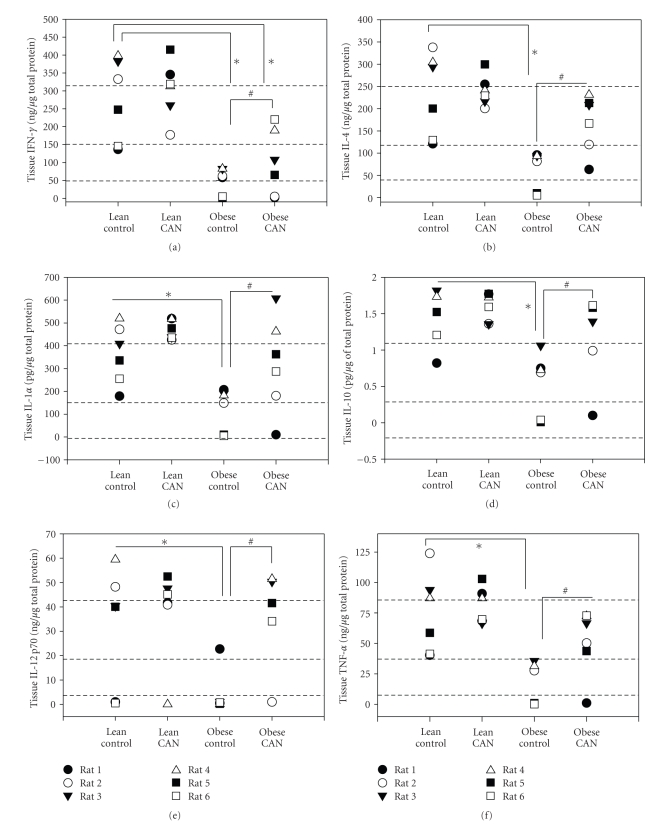
Individual kidney cytokine level (some rats undetectable) (a) interferon-*γ* (IFN-*γ*) (b) interleukin-4 (IL-4), (c) interleukin-1*α* (IL-1*α*), (d) interleukin 10 (IL-10), (e) interleukin 12p70, and (f) tumor necrosis factor *α* (TNF-*α*) in whole kidney tissue homogenate from candesartan-treated (CAN) or -untreated (c) lean and obese rats at the end of 14 weeks of treatment (*n* = 8 per body type/treatment). ∗ indicates a significant difference, *P* < .05, from lean control mean and # from obese control mean, by rank test on categorical assignments.

Surprisingly, the kidney levels of 9 out of the 14 cytokines were significantly lower in the untreated obese Zucker rats relative to lean controls. These were IFN-*γ*, IL-4, IL-2, IL-6, GM-CSF, IL-1*α*, IL-10, IL-12p70, and TNF-*α* (Figures 4 and 5). Some of these data are depicted as individual rat values (rather than means) because the protein was “below the level of detection” in some animals (Figure 5). Long-term CAN treatment increased the level of these proteins, such that they were no longer significantly different from lean control levels (except for IFN-*γ*, Figure 5(a), which remained significantly lower in CAN-treated obese rats). Similar to what was observed for MCP-1 and IL-1*β*, CAN had no effects on the levels of these 9 cytokines in the lean rats. 

Finally, a different pattern emerged for renal levels of IL-18 and GRO-KC (Figure 4). These 2 cytokines were not significantly different between lean and obese rats, but increased by CAN treatment in obese rats only, so that the levels were elevated (relative to obese control rats). No significant differences were observed for IL-5 levels between any of the groups.

### 3.6. Effects of Candesartan Therapy on eNOS and TGF-*β*

**Figure 6:**
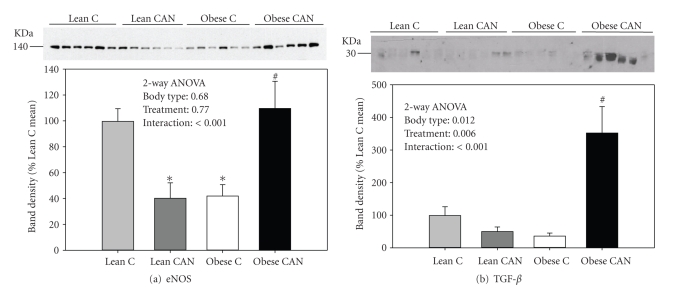
Western blotting of whole kidney eNOS and TGF-*β*. Representative immunoblot of whole kidney homogenates from -candesartan treated (CAN) or -untreated (C) lean and obese rats at the end of 14 weeks of treatment probed with (a) eNOS and (b) TGF-*β* antibodies, respectively. Equal amounts of total protein were loaded in each lane and each lane is loaded with a sample from a different rat. Below each blots is its densitometry summary (*n* = 6 rats/group). Results were analyzed by 2-way ANOVA and also by *t*-test. ∗ indicates a significant difference (*P* < .05) from lean control mean and # from obese control mean, by unpaired *t*-test.

In Figure 6, we show renal expression of endothelial nitric oxide synthase (eNOS), a protein central in oxidative-stress related pathways due to its generation of nitric oxide [38] and transforming growth factor *β*, a protein central in increased matrix formation and deposition in diabetic nephropathy [39]. Both eNOS and TGF-*β* were expressed at greatest levels in obese CAN-treated rats. Moreover, there was a significant interactive term for both proteins in that CAN reduced expression in lean rats but increased it in the obese.

## 4. Discussion

We have demonstrated that chronic candesartan treatment attenuated renal pathology and reduced renal levels of MCP-1 and IL-1*β* in obese rats. Consistently, it markedly improved renal function and lowered serum triglyceride levels in these rats. Unexpectedly, glucose tolerance was worsened. Moreover, renal levels of 11 out of 14 cytokine analyzed were in fact significantly increased by CAN in the obese rats. IL-18 and GRO-KC levels were highest in CAN-treated obese rats as compared to all other groups. Overall, our results from the cytokine array suggest that the regulation of renal cytokine levels by chronic candesartan-treatment of obese Zucker rats appeared to be primarily driven by normalization of kidney function and architecture and perhaps preservation of these necessary and sometimes protective inflammatory pathways. Decreases in serum triglyceride and/or renal MCP-1 and IL-*β* levels may have a role in the reno-protective actions of candesartan in the metabolic syndrome.

In many models of diabetes and insulin resistance, elevated RAS activity has been shown to be intimately intertwined with activation of inflammatory pathways in renal tissue [40]. In contrast to expectations, examining whole kidney homogenates, we found an increase in only two cytokines, IL-1*β* and MCP-1 in the obese Zucker rat kidneys, relative to lean controls. Increased mRNA expression of TNF-*α*, IL-1 and IL-6 has been reported in the kidney of streptozotocin-induced type 1 diabetic rats [41], a model associated with much higher levels of plasma glucose and no obesity. Less information is available with regard to renal levels of cytokines in models of the metabolic syndrome or type II diabetes. Xu et al. [42] found increased mRNA expression for 2 cytokines, MCP-1 and IL-6, in renal cortical tissue obtained from similarly aged obese Zucker rats. These levels were reduced by losartan (another ARB). In our study, we confirmed the increase at the protein level for MCP-1; however, we showed a decrease in IL-6 protein levels. In our rats, both of these cytokines were normalized by CAN. The difference between our study and that of Xu et al . [42] may have resulted from the difference in mRNA versus protein, or in the fact that we evaluated whole kidney, rather than only cortex. Another possibility is that the severity of the nephropathy may have affected the expression pattern. It is unclear whether their rats or ours had greater severity of renal disease at the time when samples were collected. 

Overall, including IL-6, we found 9 cytokines that were significantly reduced in obese versus lean rat kidney. We suggest that renal protein levels of some cytokines or chemokines may actually decline with loss of epithelial cells and the progression of renal disease. In agreement, Waldherr et al. [43] reported that TNF-*α*, IFN-*γ* and IL-2 levels in the glomeruli were undetectable in the chronic form of human glomerulonephritis, while their levels were significantly increased in the acute form of the disease. Furthermore, a study examining the relationship between the expression of IL-6 mRNA and the degree of glomerular mesangial expansion in human diabetic nephropathy demonstrated that signal intensity for IL-6 mRNA was strongest in tissues from moderate mesangial expansion but was weak in those from mild and severe mesangial expansion [44]. These and our studies support the possibility that as renal disease progresses there is decompensation at the cellular level in the immune response, perhaps due to architectural or fibrotic changes. Further studies will be needed to address this possibility. 

In further support of this hypothesis was the fact that a full 11 out of 14 cytokines in the obese rats were significantly different from lean rats in the untreated state and basically restored, or at least partially restored, by CAN. CAN attenuated renal damage, at least as gauged by reduced collagen and glycogen deposition and plasma creatinine. Furthermore, CAN had no significant effects on the lean rat cytokine profile, diminishing the possibility that other factors, for example, the fall in blood pressure with CAN as shown by us previously in both lean and obese rats [24], had any direct role on cytokine/chemokine levels. 

Nevertheless, IL-18 and GRO-KC (which were not different between untreated lean and obese) were significantly increased in the treated obese rats only. We believe that hyperglycemia and/or slower plasma glucose clearance could be responsible for increased expression of these cytokines. High glucose levels have been demonstrated to increase the secretion of both IL-18 and GRO-KC [45]. Moreover, we showed that CAN treatment increased renal expression of transforming growth factor *β*1 (TGF-*β*1), but again, only in the obese rats. A potentially causative relationship between these two variables was recently demonstrated by Bani-Hani et al. [46] in IL-18-overexpressing mice; that is, TGF-*β*1 expression was reduced when IL-18 was neutralized by antibodies. IL-18 may have a facilitative role in glucose utilization by cells. Using IL-18 knockout mice, it has been demonstrated that lack of endogenous IL-18 results in obesity, insulin resistance, and hyperglycemia [47]. Furthermore, increased serum levels of IL-18 have been associated with insulin resistance and obesity in humans [48–50].

It is nevertheless somewhat surprising that TGF-*β*1 was increased quite dramatically in the obese CAN-treated rats, relative to all other groups, while our other evidence points to reduced epithelial-to-mesenchymal transition (EMT) and the development of fibrotic renal disease in obese rats with CAN treatment. It is possible that CAN simply delays renal disease in the obese Zucker rat and that increased TGF-1*β* may be transitory and occurred at an earlier time-point in the obese control rats. It is also possible that CAN is protective at a step down-stream of TGF-*β*1 with regard to matrix accumulation. Additional studies would need to be done to clarify this matter. 

If we accept the possibility that CAN may have increased GRO-KC, IL-18, and TGF-*β*1 as a result of relative hyperglycemia in these rats, what was the mechanism for the impaired GTT? In fact, some evidence to contrary exists with regard to predicted systemic effects of AT1R blockade. ARBs, often prescribed to hypertensive subjects with the metabolic syndrome, have been demonstrated in several clinical studies to reduce the number of new onset cases of type II diabetes [51], improve glucose tolerance, and may prevent progressive beta-cell failure in diabetes [52, 53]. Tikellis et al. [53] found improved pancreatic islet morphology in Zucker diabetic fatty rats (ZDF), a substrain of the Zucker rat, that develops type II diabetes extremely early, after treatment with irbesartan (an ARB) or perindopril (an angiotensin converting enzyme inhibitor). Furthermore, candesartan was shown to improve GTT in high-fat fed Wistar rats [54]. These rats had increased expression of the peroxisomal proliferator activated receptor, subtype *γ* (PPAR-*γ*) in liver and adipose tissue, which they proposed may have been the mechanism for candesartan's effects [54].

Nonetheless, there are some clinical trials of 5 months and longer, in diabetic patients, which in agreement with our findings showed no improvement in metabolic parameters, including glucose tolerance with chronic ARB therapy [55–58]. In our study, the differences between treated- and untreated-obese rats were not large; in fact final blood glucose and insulin levels trended higher with CAN treatment, but were not statistically different and highly variable. What is clear is that they were not improved. However, surprisingly, GTT was significantly improved in the lean rats. Therefore, only obese rats responded in this somewhat negative fashion to chronic CAN therapy with regard to GTT. Glucose dose was administered intraperitoneally (ip) according to weight of the rats, but there were no significant differences in body weight between CAN-treated and control obese rats at the time of the GTT. It is possible that the lower BP in the CAN-treated rats somehow resulted in delayed uptake from the ip cavity, with subsequent delay in clearance. The blood glucose levels in the CAN-treated obese rats did not peak until 1.5 hours, while they were at their peak in the control obese rat at 30 minutes (Figure 3). However, the fall in BP in the lean rats did not affect time-of-peak for glucose, which occurred at the same time (15 minutes) for lean control and lean CAN-treated rats. It is possible that chronic CAN treatment negatively impacted the pancreatic release of insulin in the obese rats, for example, as a result of the low BP. Insulin levels were not measured during the GTT challenge; however, in the basal state they were higher (not lower) than control obese rats, suggesting relatively greater insulin resistance in these rats with the ability to compensate with hyperinsulinemia still intact. Thus, it appeared that they may have been more insulin resistant at the level of peripheral tissues. 

Candesartan-treated obese rats also exhibited relative polyuria and polydipsia, despite improvement in many histological features of the kidney, and in general renal function. Increased urine volumes could be due to glucose-induced osmotic diuresis in the CAN-treated obese rats, further supporting impaired glucose handling and insulin resistance in these rats. In contrast, lean rats treated with CAN showed the opposite; that is, they had significantly reduced urine volumes with CAN. The mechanism(s) underlying reduced urine volume with CAN treatment in these lean rats is also unknown. Ang II has been shown to stimulate thirst via AT1R in the brain. Candesartan administered peripherally has been shown to block this effect  [59]. Thus, it is possible that this is the mechanism underlying reduced water intake in the lean rats. We might speculate that this effect was masked in the obese due to thirst generated as a result of the osmotic diuresis. 

Overall, CAN therapy was protective of the kidney both functionally and histologically. CAN was able to restore or normalize (to lean levels) aberrant renal levels of 11 of 14 cytokines measured. This may be critical in the continuation of adequate immune function in the kidney of the obese rat. In contrast to obese rats, CAN had no effects on renal cytokine levels in lean rats. Moreover, these protective changes occurred despite candesartan's propensity to worsen glucose tolerance in the obese rats. Impaired GTT and increased levels of renal IL-18, GRO-KC, and TGF-*β*1 in the CAN-treated obese rats were puzzling, but of clear concern. The mechanisms underlying these effects require additional study.

## Figures and Tables

**Table 1:** General physiology^†^.

Treatment	Weight gain (g/14 weeks)	24-hour water intake (ml/d)	24-hour urine volume (ml/d)	24-hour urine volume (ml/g kidney weight/d)	Serum triglycerides (mg/ml)
Lean Control	124 ± 5	61 ± 3	48 ± 1	116 ± 4	1.44 ± 0.56
Lean CAN	119 ± 1	55 ± 1	42 ± 2*	104 ± 6	1.08 ± 0.07
Obese Control	232 ± 14*	73 ± 3*	51 ± 2	75 ± 3*	5.74 ± 0.95*
Obese CAN	191 ± 20*	107 ± 17^∗#^	69 ± 7^∗#^	107 ± 14^#^	2.66 ± 0.41^#^

Factors	Results of 2-way ANOVA for above parameters (*P*-values)				

Body Type	**<.001**	**<.001**	**<.001**	**.028**	**<.001**
Treatment	.065	.074	.174	**.024**	**.013**
Interaction	.145	**.016**	**.006**	**<.001**	**.044**

^†^mean ± sem, *n* = 7 or 8/group; *indicates a significant difference from lean control; ^#^indicates a significant difference between obese control and obese CAN groups, by unpaired *t*-test. **In bold**- *P*-values < .05 by 2-way ANOVA (significant).
